# Seasonal dynamics of megafauna on the deep West Antarctic Peninsula shelf in response to variable phytodetrital influx

**DOI:** 10.1098/rsos.140294

**Published:** 2014-11-19

**Authors:** P. Y. G. Sumida, C. R. Smith, A. F. Bernardino, P. S. Polito, D. R. Vieira

**Affiliations:** 1 Department of Biological Oceanography, Instituto Oceanográfico, Universidade de Sao Paulo, Praça do Oceanografico, 191, Sao Paulo SP 05508-120, Brazil; 2 Department of Physical Oceanography, Instituto Oceanográfico, Universidade de Sao Paulo, Praça do Oceanografico, 191, Sao Paulo SP 05508-120, Brazil; 3 Department of Oceanography, University of Hawaii at Manoa, 1000 Pope Road, Honolulu, HI 96822, USA; 4 Departamento de Oceanografia e Ecologia, Universidade Federal do Espirito Santo, Av. Fernando Ferrari, 514, Vitoria ES 29075-910, Brazil

**Keywords:** Antarctic Peninsula, megafauna, deposit feeding, time-lapse photography, elasipod holothurians, benthos

## Abstract

The deep West Antarctic Peninsula (WAP) shelf is characterized by intense deposition of phytodetritus during spring/summer months, while very little food material reaches the seafloor during winter. The response of the shelf benthic megafauna to this highly variable food supply is still poorly understood. In order to characterize the deposition of phytodetritus and the megabenthic community response, we deployed a seafloor time-lapse camera at approximately 590 m depth on the mid WAP shelf west of Anvers Island for 15 months. Seafloor photographs were taken at intervals of 12 or 24 h nearly continuously from 9 December 1999 (austral winter) to 20 March 2001 (summer) and analysed for phytodetritus deposition and megafaunal dynamics. Seafloor images indicated a marked seasonal arrival of greenish phytodetritus, with large interannual and seasonal variability in the coverage of depositing phytodetrital particles. The surface-deposit-feeding elasipod holothurians *Protelpidia murrayi* and *Peniagone vignoni* dominated the epibenthic megafauna throughout the year, frequently constituting more than 80% of the megafaunal abundance, attaining total densities of up to 2.4 individuals m^−2^. Elasipod abundances were significantly higher in summer than winter. During summer periods of high phytodetrital flux, *Pr. murrayi* produced faecal casts at higher rates, indicating intensified population-level feeding activity. In March–June 2000, faecal casts lasted longest, suggesting lower horizontal bioturbation activity during autumn–winter. Our data indicate that the *Pr. murrayi* population increases its feeding rates in response to increasing amounts and/or lability of organic matter on the sediment surface. Assuming that this species feeds on the top millimetre of the sediment, we estimate that, during periods of high phytodetrital flux, the *Pr. murrayi* population reworks one square metre of sediment surface in approximately 287 days. We suggest that *Pr. murrayi* is an important species for organic-carbon recycling on the deep WAP shelf, controlling the availability of deposited labile phytodetritus to the broader shelf benthic community.

## 2. Introduction

Oceanic and coastal Polar regions are characterized by extremes in primary productivity during an annual cycle [1–3]. Periods of high phytoplankton production may also be subject to high interannual variation related to longer term climate cycles and climate change [4,5]. Such variations in organic-matter production and phytodetritus flux may affect planktonic and benthic heterotrophic populations through changes in food availability, larval survival, recruitment rates, competition for food, feeding behaviour and other processes [6].

On the West Antarctic Peninsula (WAP) shelf, large phytoplankton blooms can occur in December–March, resulting in intense bouts of particulate organic-carbon (POC) deposition on the seafloor [7–9]. The intense spring–summer phytoplankton blooms sustain the Antarctic food web [7,10] and provide the main food source, in the form of sinking phytodetritus, to seafloor communities on the deep WAP shelf [9,11,12]. These periods of high summer production and phytodetritus flux are separated by seasons of extremely low winter (April–October) productivity, when sea-ice cover and low-light conditions prevail, allowing very little food to reach the benthos [9,13], raising questions on how benthic detritivores survive over winter months [12,14].

In the water column, where seasonal variability in food concentration is especially dramatic, grazers/detritivores exhibit varied life-history strategies. Some animals, e.g. the copepod *Paralabidocera antarctica*, store lipids to allow fasting over the winter [15,16]. Other animals, such as krill and copepods, can store lipids [6,16,17] but may also feed on ice algae and at the seafloor, or migrate to ice-free oceanic waters [18–20]. On the deep WAP shelf, benthic animals do not appear to store lipids [21,22], and yet can remain active year round [22–24], feeding on a benthic food bank of organic material stored in sediments [9,12,14,25,26].

Surface-deposit-feeding elasipod holothurians are one of the main consumers of labile phytodetritus reaching the deep WAP shelf [23,24] and other Antarctic shelf communities [14,27–29]. Elasipods have also been observed actively foraging and removing phytodetritus material in many deep-sea habitats [8,30–35]. In some abyssal areas, this foraging behaviour may limit the flux of organic material to deeper layers of the sediment column [34,36], in turn restricting the availability of freshly deposited POC to the infaunal biota.

While it has been postulated that deposit-feeding megabenthos play an important role in the flow of organic carbon through Antarctic shelf benthic food webs [28,37,38], little is known about the foraging rates of elasipod holothurians on the WAP shelf, and their response to seasonal deposition of fresh phytodetritus.

In this study, we use a time-lapse camera system deployed on the deep WAP shelf (approx. 590 m depth) to (i) monitor the arrival of phytodetritus and (ii) evaluate variations in megabenthic community structure and surface-deposit faecal production, which is used as a proxy for foraging rates, over a 15-month period at the WAP shelf floor. We find that elasipod holothurians are active throughout the year and increase in population densities through migration, modulating their feeding rates with variations in the export of particulate material from the euphotic zone. Given the high volume of sediment processed, these organisms are likely to be very important in organic-carbon cycling at the WAP shelf seafloor.

## 3. Material and methods

**Figure 1.**
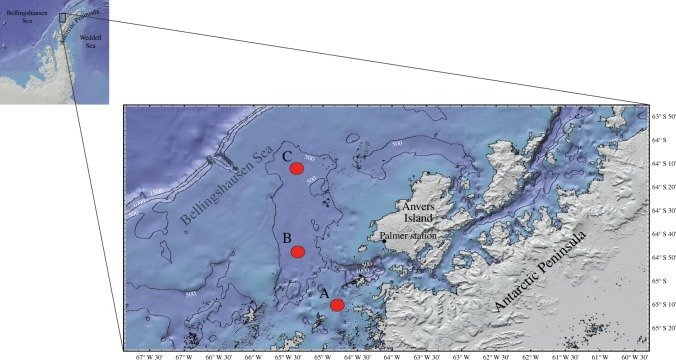
Location of the three stations (A–C) sampled on the West Antarctic Peninsula shelf during the FOODBANCS project. The time-lapse camera system was deployed at the mid-shelf station (point B).

**Figure 2.**
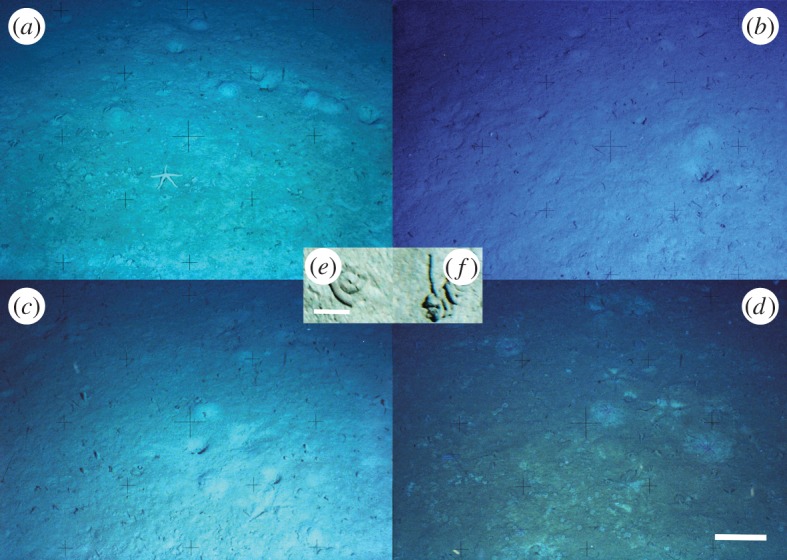
Seafloor photographs taken at four different periods in the West Antarctic Peninsula (WAP) shelf Station B. (*a*) Picture taken in the November 1999–March 2000 period. Note the presence of a thin layer of phytodetritus at the seafloor. (*b*) March 2000–June 2000. During this period, no phytodetritus was found. (*c*) In June 2000–October 2000, no phytodetritus layer was observed. (*d*) November 2000–March 2001. Dense carpets of phytodetritus (approx. 2 cm thick) were found over the seafloor. Inset: faecal casts of the two main elasipod holothurians on the WAP shelf: (*e*) *Pr. murrayi* and (*f*) *Pe. vignoni*. The scale bar of the larger picture represents 20 cm. In the inset, the scale bar is 2 cm.

A film-based time-lapse photographic camera system Photosea 2000 with two 150 J Photosea 1500s strobes and a DeepSea Power & Light 24 V SeaBattery was deployed during the FOODBANCS (FOOD for the Benthos on the ANtarctic Continental Shelf) project at mid-shelf station B (64° 48.00^′^ S, 65° 21.30^′^ W; *ca* 590 m depth) for five contiguous periods of three months (table 1; see Smith & DeMaster [26] for a review on the FOODBANCS programme) on the WAP shelf (figure 1). The system took photographs at 12 or 24 h intervals (table 1) during a 15-month period between 8 November 1999 and 20 March 2001 (figure 2) [26]. The camera was mounted on an aluminium tripod at an elevation of 152 cm above the seafloor and took pictures at a 45° angle of *ca* 4 m^2^ of seafloor. All deployments used Kodak 5279 Vision 500 T motion picture film (ASA 500). The camera malfunctioned between 19 January 2000 and its recovery on 9 March 2000, and between 25 September 2000 and 25 October 2000, so there were no photographs during these intervals. In addition, no photographs were collected during the *ca* 10-day periods between recovery and redeployment during mid-project cruises. At each deployment/recovery, at least one otter trawl sample was taken to capture local megafauna for identification and reproductive studies [23].

**Table 1.** Sampling period and photographic coverage of FOODBANCS programme. FB I–V are the FOODBANCS cruises. Time-series photographs were collected between cruises.

	period	station ID	deployed	recovered	frame interval (h)	days of good coverage^*a*^
FB I–II	Nov 99–Mar 00	CRS 481	7 Dec 1999	9Mar 2000	12	28
FB II–III	Mar 00–Jun 00	CRS 562	18 Mar 2000	15 Jun 2000	12	88
FB III–IV	Jun 00–Oct 00	CRS 635	19 Jun 2000	27 Oct 2000	24	97
FB IV–V	Nov 00–Mar 01	CRS 716	3 Nov 2000	2 Mar 2001	12	121

^*a*^Number of continuous days with appropriate light conditions, i.e. proper functioning of the flash, that allowed analysis of epifauna.

In the laboratory, images were digitalized using a Nikon Super Coolscan 5000ED 35 mm/APS (IX240) film scanner into tagged image file format (TIFF) for later analysis. The automatic colour compensation provided by the scanner was reversed to recover the original slide colours. The reversal process used the black frame as a reference. Each of the original 1008×672 pixel TIFF images was converted to four (RGBI) matrices, centred through a pattern-recognition Matlab program that identified the position of the central crosshatch in the Photosea 2000 image. An area of 800×620 pixels centred on the crosshatch was kept and outside areas cropped from all images. A Canadian perspective grid (50×50 cm squares) [39] was then constructed based on the camera elevation and angle relative to the seafloor and superimposed on each oblique image using Matlab. As the oblique grid cells represented squares in the seafloor, the edges of a larger square (1×1 m) formed by the four bottom centre cells were used for a perspective transformation. This transformation places the image viewpoint at a right angle from the seafloor (assumed to be planar) allowing distances and areas to be calculated. The quantitative part of the study focused on the epibenthic megafauna living on soft sediments. During the course of the study the camera never faced hard bottoms. Owing to different lighting conditions and to minimize perspective correction errors, only the well-illuminated bottom area of the images (1.6 m^2^ or 491×491 pixels) was used in the analysis, thus reducing errors in the counting and identification of species [40]. We could resolve and identify animals exceeding approximately 2 cm in minimum dimension.

Epifaunal organisms were counted in each image (*N*=452) and numerical density presented as individuals per square metre. The number of times a single individual appeared in consecutive images was recorded in order to evaluate the activity of benthic organisms (mostly holothurians). Individuals were visually identified by their size, position in the frame and bioturbation track in consecutive frames. Holothurian movement rates were calculated as the average linear distance of single individuals between consecutive frames (12 h). As these animals move irregularly, distances represent minimal movement rates and are likely to be underestimated, as we could not estimate the distance travelled (or direction of movement) from individuals that only appeared once in photographs.

Faecal-cast numerical densities and persistence times were calculated for the entire period for the holothurian *Protelpidia murrayi*, which produced the most conspicuous faecal casts (figure 2). Faecal casts appearing in images were marked and tracked in each successive photograph until they could no longer be resolved. For each cast, length (i.e. the uncoiled faecal cast) and width (i.e. the diameter of a faecal-cast section) were measured and the volume of sediment processed was calculated assuming the faecal casts to be cylindrical. Individual faecal-cast production was estimated for individuals that could be tracked in pictures. Population-level faecal production was calculated using the total volume of faeces produced per day in a given area. Faecal-cast parameters were compared using either one-way ANOVA or Kruskal–Wallis test after testing for the homogeneity of variances. Differences were considered significant at *p*<0.05. When significant differences were found, we applied the *post hoc* tests of Tukey or Dunn depending on the assumptions of normality of data (Bartlett's test).

Sediment reworking time for individual *Pr. murrayi* was calculated assuming that animals feed on the top millimetre of the sediment surface based on observations of animals kept in the laboratory. Therefore, total reworking of the top millimetre of 1 m^2^ of sediment implies 1000 cm^3^ of sediment ingestion. The total reworking time of *Pr. murrayi* population at a given season was then calculated dividing 1000 cm^3^ m^−2^ by the production of faecal casts per day. Therefore, this measurement represents the time of foraging required to rework a square metre of sediment. Seasonal ingestion rates were also tested by ANOVA or Kruskal–Wallis tests at a significance level of 0.05 and the same *post hoc* tests applied as explained above. Data on faecal casts and reworking times were compared with POC fluxes obtained by Smith *et al.* [9] during the same period.

## 4. Results

### 4.1 Epibenthic megafauna composition, numerical density and size

Echinoderms constituted the vast majority of the epibenthic megafauna at the WAP shelf station throughout the study period. The elasipod holothurians *Pr. murrayi* and *Peniagone vignoni* were the most abundant throughout the year, with lower densities during winter months (June 2000 to October 2000; table 2). *Protelpidia murrayi* represented over 52% of the epibenthic megafauna during spring/summer months, whereas *Pe. vignoni* had two peaks of abundance during the austral autumn/winter and summer of 2000–2001 (table 2). Other echinoderms included the sea stars *Psilaster charcoti*, *Henricia* spp., and an unidentified species, as well as the sea urchins *Amphipneustes* spp. and *Ctenocidaris perrieri*. Crinoids and ophiuroids were observed occasionally, as were molluscs, including the octopus *Pareledone charcoti* and the gastropod *Harpovoluta charcoti*. Giant nemerteans (*Parborlasia corrugatus*) and fish were also present in small numbers. A number of species present in pictures, including small penatulaceans and shrimps, were not counted because they were difficult to identify and the densities were very low.

**Table 2.** Benthic megafauna density (individuals m^−2^) from time-lapse photographs during the FOODBANCS project.

	mean density (s.d.) (individuals m^−2^)	minimum and maximum density (individuals m^−2^)	per cent of total
FB I–II (Nov 99–Mar 00)			
*Pr. murrayi*	0.34 (0.36)	0/1.08	72.1
*Pe. vignoni*	0.04 (0.12)	0/0.43	8.3
*Amphipneustes* spp.	0.03 (0.10)	0/0.43	6.5
Asteroidea	0.06 (0.11)	0/0.43	13.1
total megafauna	0.48 (0.42)	0/1.52	
FB II–III (Mar 00–Jun 00)			
*Pr. murrayi*	0.22 (0.34)	0/1.08	45.7
*Pe. vignoni*	0.22 (0.31)	0/1.52	46.1
Echinoidea	0.02 (0.11)	0/0.43	4.5
Asteroidea	0.02 (0.07)	0/0.43	3.7
total megafauna	0.48 (0.43)	0/1.52	
FB III–IV (Jun 00–Oct 00)			
*Pr. murrayi*	0.06 (0.15)	0/0.43	26.3
*Pe. vignoni*	0.08 (0.18)	0/0.87	36.5
*Amphipneustes* spp.	0.03 (0.08)	0/0.52	11.4
*C. perrieri*	0.02 (0.05)	0/0.52	6.8
Asteroidea	0.04 (0.14)	0/0.58	19.0
total megafauna	0.23 (0.29)	0/0.87	
FB IV–V (Nov 00–Mar 01)			
*Pr. murrayi*	0.38 (0.33)	0/1.73	52.7
*Pe. vignoni*	0.26 (0.36)	0/1.96	36.1
*Amphipneustes* spp.	0.02 (0.08)	0/0.43	2.6
Asteroidea	0.06 (0.13)	0/0.65	7.9
Mollusca	0.004 (0.05)	0/0.52	0.7
total megafauna	0.71 (0.54)	0/2.39	

**Figure 3.**
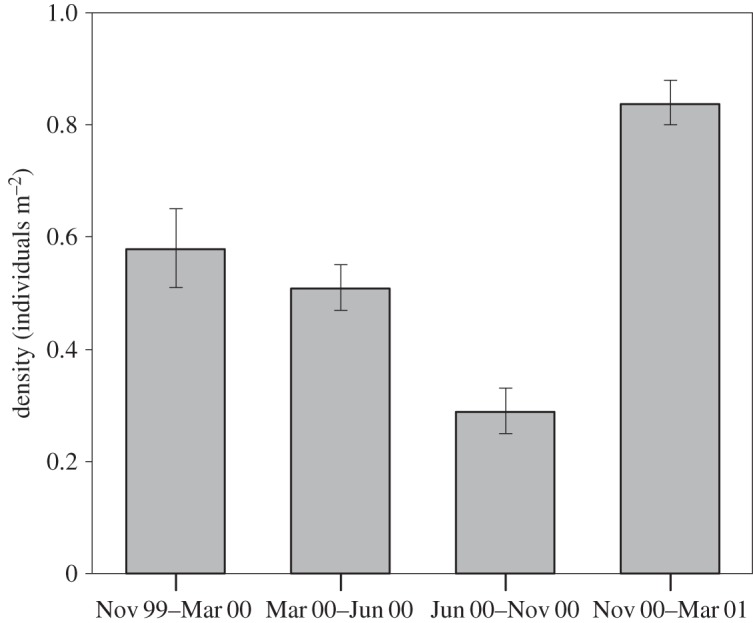
Mean epibenthic megafaunal density (individuals m^−2^) measured over the study period. Vertical lines are ±1 s.e.

The full time series showed that there was a marked increase in the number of epibenthic megafauna towards the end of the study period (figure 3). Total megafaunal densities were highest during the summer months, especially in November 2000–March 2001 (table 2), when numbers were significantly higher than in all other intervals (Tukey test, *p*<0.05). During this period, megafaunal numbers reached peaks of 2.4 individuals m^−2^. The lowest mean densities (less than 50% of spring/summer) were observed during winter months (June 2000–October 2000; table 2; Tukey test, *p*<0.05).

**Figure 4.**
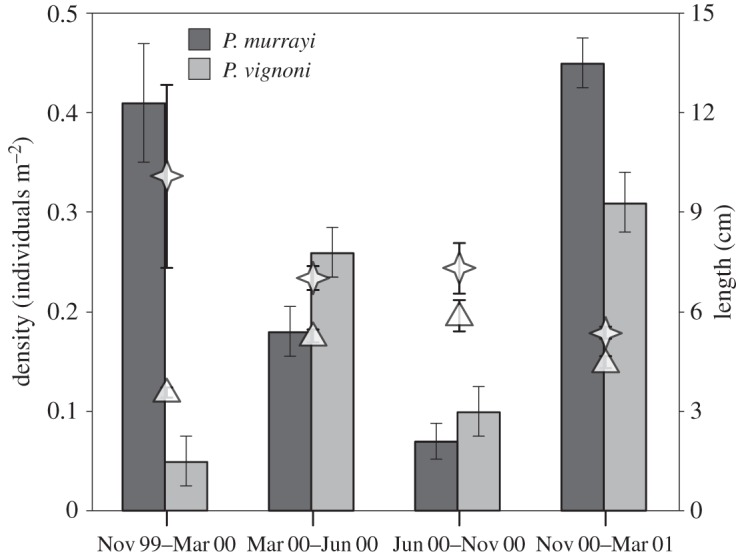
Numerical density and size of elasipod holothurians in time-lapse photographs. Bars represent numerical density; triangles and stars are mean lengths of the elasipods *Pr. murrayi* and *Pe. vignoni*, respectively. Vertical lines are ±1 s.e.

The two most numerous megafaunal species, *Pr. murrayi* and *Pe. vignoni*, exhibited significant temporal variations in abundance. The density of *Pr. murrayi* was significantly lower during June 2000–October 2000 (0.07±s.d. 0.02 individuals m^−2^) than in the remaining sampling periods (maximum in November 2000–March 2001 of 0.45±s.d. 0.03 individuals m^−2^; Tukey test, *p*<0.01). *Protelpidia murrayi* individuals were larger during June 2000–October 2000 (mean length 5.9 cm) than in November 1999–March 2000 (3.6 cm; Tukey test, *p*<0.01; figure 4). The density of *Pe. vignoni* ranged from 0.05 to 0.26 individuals m^−2^ (figure 4), with densities in November 1999–March 2000 significantly lower than in November 2000–March 2001 (Tukey test, *p*<0.01). The mean size of *Pe. vignoni* individuals was significantly smaller in November 2000–March 2001 (5.4±s.d. 2.2 cm) than in the first two periods (10.1±s.d. 2.8 cm and 7.0±s.d. 0.4 cm, respectively; Tukey test, *p*<0.01; figure 4).

### 4.2 Movement rates and feeding dynamics

Individual holothurian movement, based on the average linear distance travelled by single individuals between consecutive 12 h interval frames (*N*=2 to 14 individuals), showed that *Pr. murrayi* individuals had similar seasonal movement rates (1.1–2.0 cm h^−1^), whereas *Pe. vignoni* individuals moved almost twice as fast during winter months compared with November 2000–March 2001 (Tukey test, *p*<0.01; table 3). Movement rates of *Pe. vignoni* were higher than *Pr. murrayi* during winter (Tukey test, *p*<0.01; table 3) but did not differ during the summer bloom period of November 2000–March 2001 [9]. We observed no influence from animal size on movement rates, but note that our sample size was small.

**Table 3.** Seasonal average movement rates for *Protelpidia murrayi* and *Peniagone vignoni*based on individuals identified on consecutive time-lapse photographs.

	*N*	mean size (s.d.) (cm)	mean movement rate (s.d.) (cm h^−1^)
Nov 99–Mar 00			
*Protelpidia*	8	3.2 (0.6)	1.1 (0.3)
*Peniagone*	n.a.		
Mar 00–Jun 00			
*Protelpidia*	17	4.5 (1.2)	2.0 (1.4)
*Peniagone*	15	7.2 (4.1)	3.5 (1.8)
Jun 00–Oct 00			
*Protelpidia*	7	4.1 (2.4)	1.3 (0.9)
*Peniagone*	2	6.3 (3)	2.7 (0.1)
Nov 00–Mar 01			
*Protelpidia*	43	5.5 (3.3)	1.9 (1.7)
*Peniagone*	26	5.1 (1.4)	1.7 (1.5)

**Figure 5.**
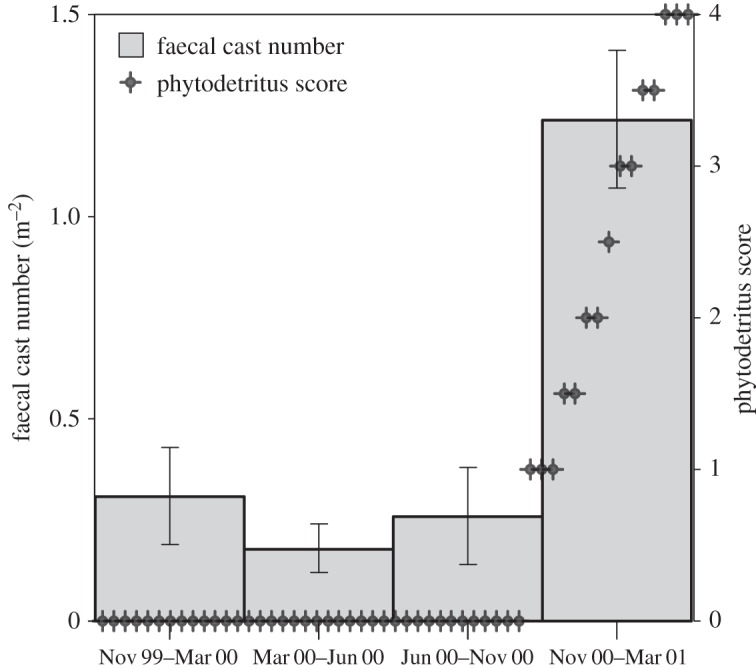
Mean number of faecal casts deposited on the seafloor by the holothurian *Pr. murrayi* pooled per sampling period. The right *Y* -axis represents scores for phytodetritus coverage of the seafloor as follows: no greenish, flocculent phytodetritus visible at the seafloor (*score*=0); diffuse, greenish, flocculent material visible in some areas but bioturbation traces readily visible (score 1); much of the seafloor (50–90%) covered with greenish phytodetritus, with bioturbation traces partially filled (*score*=2); more than 90% phytodetritus cover (*score*=3) [9]. Score 4 represents dense, up to 2 cm thick phytodetritus carpets. Vertical lines are ±1 s.e.

The average faecal-cast volume for both holothurians varied from 1.8 to 6.5 cm^3^ and was only significantly different between November 1999–March 2000 and March 2000–June 2000 (Dunn test, *p*<0.05). Faecal-cast volume was positively correlated with animal length for *Pr. murrayi* (*N*=190; Pearson *r*=0.5468, *p*<0.001). We observed no marked trend in the number of faecal casts with time until the end of January 2001, when larger quantities of faecal casts were found (figure 5). This followed closely the amount of phytodetritus found on the surface of sediments (figure 5) [9]. The mean number of faecal casts of *Pr. murrayi* averaged over each sampling period was significantly different, with the highest mean during the November 2000–March 2001 period (Dunn test, *p*<0.05). The average density of faecal casts during this period (1.3±1.9 s.d. faecal cast m^−2^) was twice as high as that measured in the previous summer (0.6±1.0 s.d. faecal cast m^−2^), when no phytodetritus carpet was evident. During the winter period, the mean number of faecal casts was approximately three to five times lower (0.4±s.d. 0.8 faecal cast m^−2^ for March 2000–June 2000 and 0.3±s.d. 0.8 faecal cast m^−2^ for June 2000–October 2000). Also, during the first three sampling periods November 1999–October 2000, many frames had no freshly deposited faecal casts (figure 5). On the other hand, *Pr. murrayi* individuals deposited new faecal casts almost continuously from November 2000 to March 2001 (figure 5).

**Figure 6.**
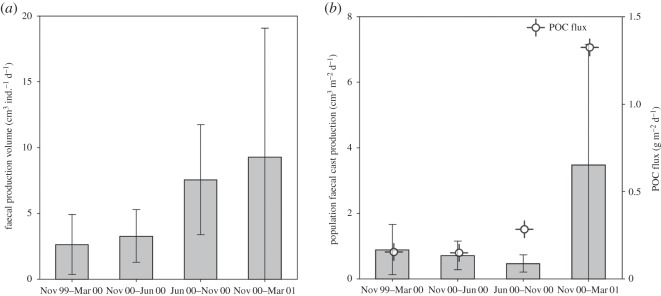
Mean faecal-cast volumetric production rate per individual (*a*) and population-level faecal-cast production rate (*b*) for the holothurian *Pr. murrayi*. Data were pooled for each sampling period. Also shown in (*b*) is the POC flux in the study area collected during the same period by Smith *et al.* [9]. Vertical lines are ±1 s.e.

Production of faecal casts by *Pr. murrayi* at both individual and population levels was significantly higher from June 2000 to March 2001, especially during the phytodetritus bloom (Dunn test, *p*<0.05; figure 6*a*,*b*). Average faecal-cast production rates per individual varied seasonally (figure 6*a*) with an increase in faecal production in June 2000–November 2000, when phytodetrital flux was low. Although *Pr. murrayi* were on average largest during the March 2000–October 2000 interval (figure 4), per individual faecal-cast production was highest during the November 2000–March 2001 bloom months, which had smaller *Pr. murrayi* (figure 6). The average persistence time of faecal casts on the seafloor was lowest in the summer of 1999–2000 (mean of 135 h) and highest (317 h) in the subsequent winter (March 2000–June 2000; Dunn test, *p*<0.05). During the high phytodetritus flux period (November 2000–March 2001), mean faecal persistence time was approximately 189 h. In general, higher individual faecal-cast volume was positively correlated with faecal-cast persistence time (*N*=292; Pearson *r*=0.3816, *p*<0.001).

**Figure 7.**
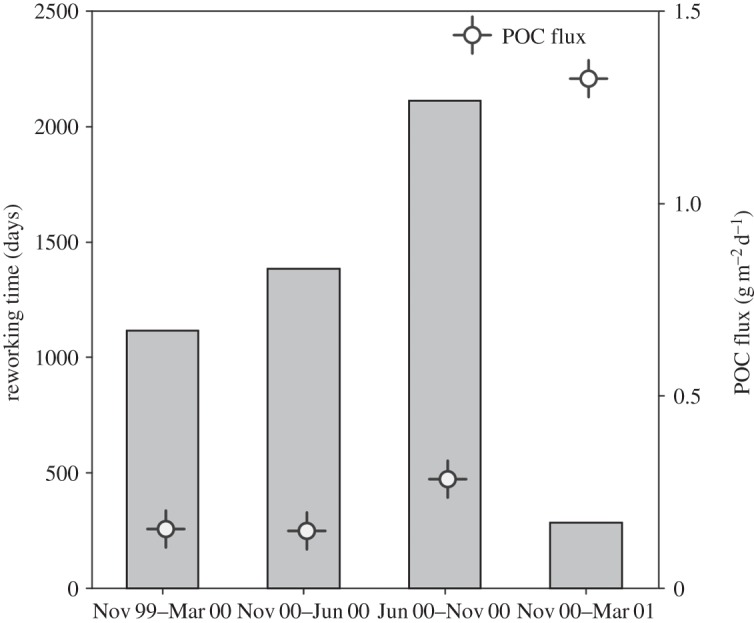
*Protelpidia murrayi* population-level sediment reworking times for the top millimetre layer of sediment on the WAP shelf. POC flux data were collected for the same study area and period by Smith *et al.* [9].

Population-level faecal-cast production rates (surface-sediment reworking rates) for *Pr. murrayi* varied across seasons, being highest during the period of high POC flux and phytodetritus concentration (figures 6 and 7). During November 2000–March 2001, population-level faecal production rates exceeded faecal production rates in all previous periods by a factor of 3.5 (Dunn test, *p*<0.05; figure 6*b*). In addition, surface-sediment reworking times in November 2000–March 2001 were 1/7 of those in winter/spring 2000, with the equivalent of the top 1 mm of sediment being reworked during the period of high phytodetrital availability with approximately 287 days (figure 7).

## 5. Discussion

Our time-lapse photographic record shows evidence of strong interannual and seasonal variability in deposition of phytodetritus over the WAP shelf, associated with significant changes in megafaunal abundance. This high variability is consistent with previous studies documenting substantial seasonal and interannual variability in the flux of particulate organic matter (POM) and chlorophyll *a* to the WAP shelf sediments [7,9,10,12,41,42]. The time-lapse images revealed no phytodetrital coverage of sediments from the summer of 1999–2000 until the winter of 2000, which was followed by a rapid (i.e. days) increase in detritus accumulation in November 2000 [9]. The high interannual variability of phytodetrital accumulation may result from complex pelagic nutrient dynamics and variable ice retreat controlling phytoplankton blooms, variable grazing by zooplankton and lateral transport of surface phytoplankton-rich waters over the WAP shelf [43–45]. During summer periods of high primary productivity, POM fluxes are enhanced by the formation of larger, heavier marine snow aggregates and zooplankton faecal pellets [46,47]. During the spring–summer of 2001, thick carpets (approx. 2 cm) of phytodetritus were observed on the seafloor and appear to have provided an important food supply to the deposit-feeding megafauna [9,12,45].

Previous studies have indicated that temporal variability in the abundance and/or activity of macro- and megabenthos over a 15-month period on the deep WAP shelf was largely decoupled from phytodetritus accumulation and POC flux at the seafloor [24]. The weak coupling between seasonal POC flux and megafaunal and macrofaunal communities in the WAP shelf has been postulated to result from the presence of a sediment ‘food bank’ for deposit feeders [12,24,26,45]. The time-lapse images give further support for the food bank hypothesis, revealing that holothurians forage across the seafloor (i.e. inferred from movements), and consume and egest sediments, even during winter periods of very low food flux to the WAP shelf floor.

The community dominant *Pr. murrayi* fed continuously on surface sediments throughout the year, as indicated by the production of faecal casts during all our study intervals (figure 5). Similar continuous feeding on the labile organic matter available in surface sediments and on fresh phytodetritus deposited on the seafloor during the spring/summer season has been documented for infaunal echiurans and holothurians on the WAP shelf [12,23,26]. These finding indicate the presence of a sediment food bank on the WAP shelf fuelled by summer blooms, which may persist due to low bacterial organic-matter degradation rates in the extremely cold Antarctic shelf temperatures [9,12].

Although *Pr. murrayi* does feed year round, this abundant surface-deposit feeder does appear to modulate its sediment ingestions rates in response to increased food availability at the seafloor, as suggested by Sumida *et al.* [24]. This response is indicated by a nearly fourfold increase in the production of faecal casts per individual during the summer of 2000–2001 in comparison with the previous summer, leading to diminished gut residence times presumably owing to the higher food quality [9,45,48,49]. *Protelpidia murrayi*, which is relatively unselective during particle ingestion [25], relies on selective digestion of organic matter; the presence of higher concentrations of labile phytodetrital material may allow it to pass sediment through the gut at higher rates, while maintaining adequate digestive yield [50,51].

At the *Pr. murrayi* population level, the production of faecal casts is seven times higher during summer of 2000–2001 than the previous summer, as a consequence of higher population densities probably resulting from immigration, larger body sizes and higher individual-level feeding rates. These variations in *Pr. murrayi* population densities and faecal-cast production rates yield substantial temporal variation in reworking times for the top millimetre of the sediment, with a turnover time approximately 287 days during the period of intense phytodetrital accumulation and approximately 2114 days when fluxes were lowest in winter months (figure 7).

Faecal-cast duration suggests that the benthic community as a whole passes through cycles of low and high bioturbation rates, with faecal casts persisting approximately twice as long (approx. 317 h) during winter months than during summer periods. Megafaunal movement rates and foraging activities are likely to be the main cause of the disappearance of faecal coils in this low-flow environment [52]. We found no evidence of sediment resuspension in our images, which suggests that currents are not important in erasing megafaunal traces and faecal casts from the sediment surface. These cycles of bioturbation activity may also be important in the vertical mixing of POM across the sediment–water interface [53]. Such vertical transport will make food available to subsurface feeders and can ultimately lead to carbon burial. For example, the Antarctic sea urchin *Abatus ingens* reworks the top 2 cm of shallow-water sediments 2 to 17 times per year, influencing a variety of sediment processes including nutrient mixing and oxygen penetration to lower layers [54]. In our study area, faecal-cast duration seems likely to be controlled by a combination of ‘retracking’ by epifauna and diffusive sediment mixing by infauna (cf. Wheatcroft *et al.* [52]). The higher population-level sediment reworking rates for *Pr. murrayi* during the period of high phytodetritus availability suggests that ‘retracking’ by epibenthos contributed to the reduction in faecal-cast duration during November 2000–March 2001.

Because of high population densities and high feeding and faecal-cast production rates, *Pr. murrayi* may be geochemically important [55], modulating the distribution of labile organic matter at the sediment–water interface, and within the sediment column, on the deep WAP shelf. During periods of high phytodetrital flux, the *Pr. murrayi* population appears capable of processing the top 1 mm of sediment, and the recently deposited phytodetritus, on 200–300 day time scales, i.e. over time scales roughly similar to the spring–summer bloom period and the occurrence of phytodetritus at the seafloor [9]. As a consequence, much of the labile phytodetritus depositing on the WAP floor during the summer bloom may pass through a holothurian ‘filter’, becoming assimilated or redistributed as faecal casts by surface-deposit-feeding holothurians before becoming available to the broader deposit-feeding community. Thus, the dynamics of deposit-feeding megafauna, in particular *Pr. murrayi*, should be included in models of biogeochemical cycling and climate change on the WAP shelf. Because surface-deposit-feeding elasipod holothurians similar to *Pr. murrayi* are abundant globally at bathyal depths and have been shown to actively consume labile phytodetritus [56], these holothurians are likely to modulate the distribution of labile organic matter on many continental margins.

## Data accessibility

Data available from the Dryad Digital Repository: http://doi.org/10.5061/dryad.ks515.
