# Seasonal dynamics of megafauna on the deep West Antarctic Peninsula shelf in response to variable phytodetrital influx

**DOI:** 10.1098/rsos.140464

**Published:** 2014-12-17

**Authors:** P. Y. G. Sumida, C. R. Smith, A. F. Bernardino, P. S. Polito, D. R. Vieira

*R. Soc. open sci.* **1**, 140294. (Published online 19 November 2014). (doi:10.1098/rsos.140294)

Following publication of our article [1], it has come to our attention that there is an error in the second to last sentence of the abstract.

The sentence in the published article reads:

‘Assuming that this species feeds on the top millimetre of the sediment, we estimate that, during periods of high phytodetrital flux, the *Pr. murrayi* population reworks one square metre of sediment surface in approximately 287 days’.

The sentence should read as follows:

‘Assuming that this species feeds on the top millimetre of the sediment, we estimate that, during periods of high phytodetrital flux, the *Pr. murrayi* population reworks the entire sediment surface in approximately 287 days’.

This error was introduced by the authors during the proofing stage. We apologize for any inconvenience caused.
